# Case Report: Management of Traumatic Carotid-Cavernous Fistulas in the Acute Setting of Penetrating Brain Injury

**DOI:** 10.3389/fneur.2021.715955

**Published:** 2022-02-11

**Authors:** Andrea Loggini, Tareq Kass-Hout, Issam A. Awad, Faten El Ammar, Christopher L. Kramer, Fernando D. Goldenberg, Christos Lazaridis, Ali Mansour

**Affiliations:** ^1^Neurosciences Intensive Care Unit, Department of Neurology, University of Chicago Medicine and Biological Sciences, Chicago, IL, United States; ^2^Section of Neurosurgery, Department of Surgery, University of Chicago Medicine and Biological Sciences, Chicago, IL, United States

**Keywords:** penetrating brain injury, endovascular intervention, traumatic carotid cavernous fistula, traumatic cerebrovascular injury, neurocritical care

## Abstract

Traumatic carotid-cavernous fistulas (tCCFs) after penetrating brain injury (PBI) have been uncommonly described in the literature with little guidance on optimal treatment. In this case series, we present two patients with PBI secondary to gunshot wounds to the head who acutely developed tCCFs, and we review the lead-up to diagnosis in addition to the treatment of this condition. We highlight the importance of early cerebrovascular imaging as the clinical manifestations may be limited by poor neurological status and possibly concomitant injury. Definitive treatment should be attempted as soon as possible with embolization of the fistula, flow diversion via stenting of the fistula site, and, finally, vessel sacrifice as possible therapeutic options.

## Introduction

Traumatic carotid-cavernous fistulas (tCCFs) represent abnormal vascular shunting between the carotid artery, in its cavernous segment, and the cavernous sinus, after direct or indirect trauma (1). tCCFs have a prevalence ranging between 0.2 and 4% in closed brain injury and are typically associated with a basilar skull fracture (2, 3). Common clinical manifestations include proptosis, chemosis, orbital bruits, headache, stroke symptoms, and visual disturbances (1). tCCF clinical syndrome can develop rapidly post injury, though it may take a few days to weeks to become symptomatic (4–6). Sporadic cases have also been reported of tCCFs detected years after the initial injury (6, 7). CCFs are divided into high and low flow lesions. Barrow et al. defined four types (A-D) of CCFs based on the arterial connection to the cavernous sinus. Type A CCFs are direct, high-flow lesions, directly connecting the internal carotid artery (ICA) to the cavernous sinus. Type B-D CCFs are low-flow lesions. Type B CCFs arise from meningeal branches of the external carotid artery (ECA); type C CCFs arise from meningeal branches of the ICA; whereas type D CCFs arise from meningeal branches of both ICA and ECA (1, 8). Among the four subtypes, high flow shunts, or type A fistula per Barrow classification system (8), require more urgent repair as, if left untreated, they have a higher risk to progress causing arterialization of the cavernous sinus and intracerebral veins, and intraparenchymal hemorrhage (9). With the advent of endovascular approaches, endovascular treatment is feasible and preferred over open surgical repair (6, 10–12).

Conversely, low flow tCCFs tend to clinically manifest in a more subacute manner, predominantly presenting with gradually progressive ocular symptoms characterized by conjunctival injection, proptosis, and ophthalmoparesis. Treatment of low-flow tCCFs is endovascular, mostly performed electively, after the acute phase of trauma (1).

In penetrating brain injury (PBI), specifically gunshot wounds to the head, the presence of tCCFs has only been described in a few case series (4, 13–15). The current guidelines for the management of penetrating brain injury, now more than two decades old, recommend treatment with either a surgical or endovascular approach (16). However, there exists no guidance on the timing of the repair, ideal approach, or possible complications related to the respective interventions. Unique to tCCFs associated with gunshot wounds is the potential risk of exsanguination or bleeding into the brain proper; this is related to the fact that oftentimes in such injuries the cavernous sinus dura and the adjacent bone are disrupted by the projectile. Furthermore, the management of tCCF in the GSW population is particularly relevant as gunshot patients represent a unique challenge be it due to the presence of concomitant cranio-cervical vascular injury, other organ involvement, or contraindications for anticoagulation and /or antithrombotic use (17).

In this case series, we describe two patients with gunshot wounds to the head who acutely developed tCCFs. We discuss the specific challenges to timely diagnosis, evaluation, and treatment of this condition, and we review the need for practical recommendations pertaining to the management of this unique population.

## Case Presentation

### Case A

**Figure 1:**
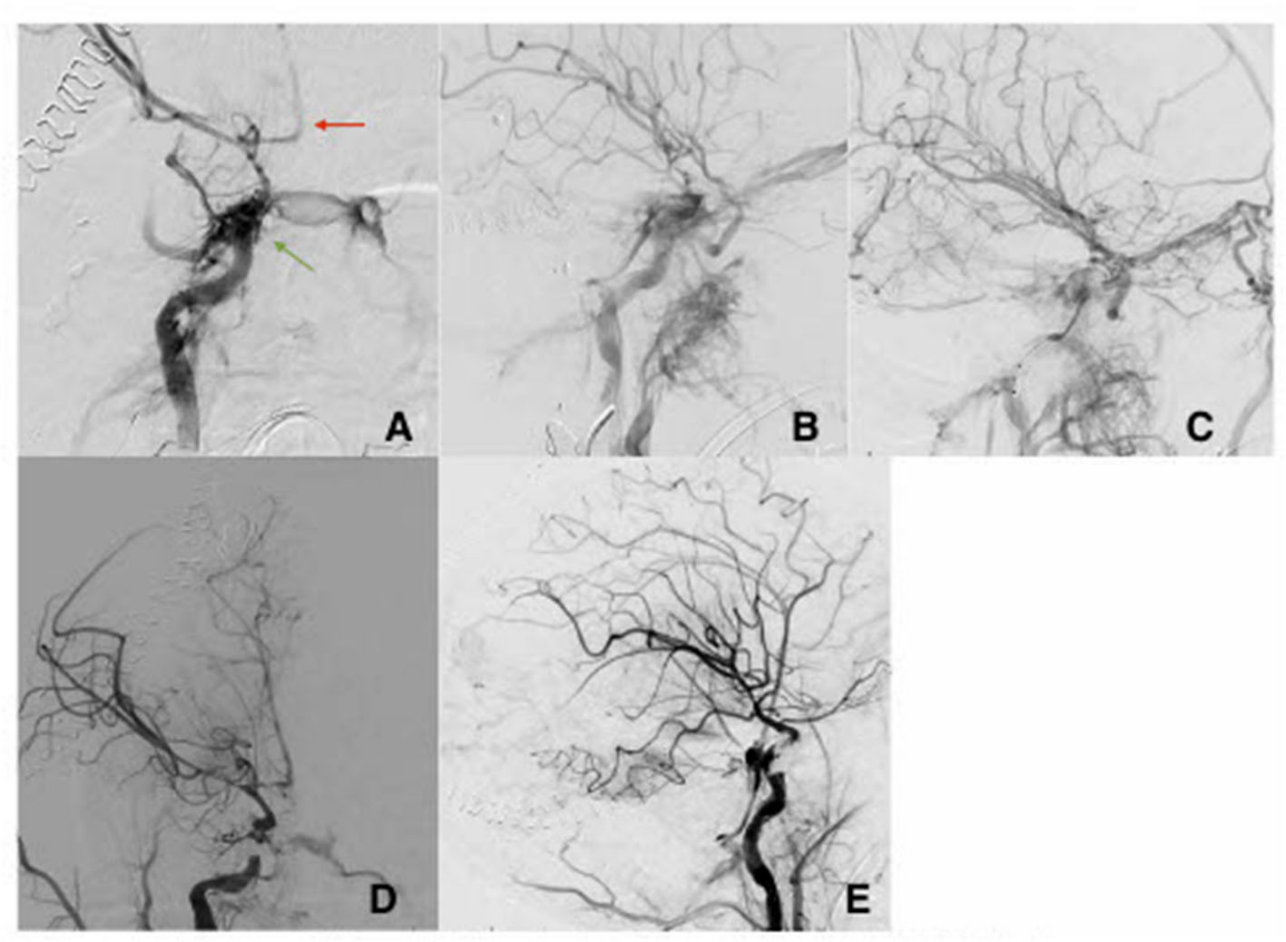
AP view **(A)** and lateral view **(B)** with direct fistula between cavernous segment of the right ICA and the cavernous sinus (green arrow). There is evidence of severe vasospasm in the intracranial vascular tree (red arrow). **(C)** Oblique view post partial coiling of the fistula site. **(D)** AP view and lateral view **(E)** of the right ICA post flow diverting stents and coiling with improvement of the CCF and improved flow intracranially.

Patient A is a 23-year-old female who presented to the emergency department after a gunshot wound to the right side of the head with dural penetration. Her initial Glasgow Coma Scale (GCS) was 7 (E1, V1, M5), prompting urgent intubation. Computerized head tomography (HCT) demonstrated a right temporal lobe penetrating injury with retained bullet fragment, a traumatic subarachnoid hemorrhage in the basal cisterns, diffuse cerebral edema, and a 5 mm right to left midline shift at the foramen of Monroe. Extensive skull fractures were noted along the course of the bullet including the right maxillary sinus, the floor of the middle cranial fossa, and the mastoid portion of the temporal bone. Calvarial fractures extended superiorly through the right greater sphenoid wing to the coronal suture, as well as posteriorly through the right parietal bone to the lambdoid suture. The fracture over the right sphenoid tracked across the skull base to involve the carotid canal. Numerous metallic fragments were retained intracranially including a large 9 mm bullet fragment lodged between the right temporal lobe and parietal lobes. Computerized Tomography angiogram of the neck and head (CTA head and neck) identified an irregularity of the right Internal Carotid Artery (ICA) in the proximity of the carotid canal fracture, concerning vascular injury. Over the subsequent hours, the patient underwent emergent decompressive hemicraniectomy and a right subgaleal drain was placed. Three days later, as hemodynamic stability was demonstrated, conventional cerebral angiography was done revealing a high-flow right tCCF with significant arterialization of cortical veins. The tCCF was embolized via a transvenous approach with a significant reduction of the fistulous flow (Figures 1A–C). Seven days after the embolization, the patient developed fixed mydriasis of the right pupil. Repeat conventional cerebral angiography demonstrated preserved patency of tCCF now with prominent flow through the right superficial ophthalmic vein, bilateral cavernous sinus, pterygoid plexus, and inferior petrosal sinus. Following a multidisciplinary discussion, a flow-diverting stent was deployed across the fistula in the cavernous segment with a subsequent significant reduction in arteriovenous shunting (Figures 1D,E). The benefit of dual antiplatelet therapy was believed to outweigh the risk of worsening intracranial hemorrhage and bleeding in the setting of recent hemicraniectomy. The patient remained in the hospital for 51 days and suffered multiple neurological complications, including cerebral vasospasm, development of a pseudoaneurysm in the right anterior choroidal artery that was embolized, and hydrocephalus, requiring ventriculo-peritoneal shunting (VPS). The use of dual antiplatelet therapy was maintained throughout her hospitalization; this presented a significant challenge to the management of the pseudoaneurysm, the placement of VPS, and eventually the performance of tracheostomy and percutaneous gastrostomy. She was discharged to a long-term acute care facility. Her GOSE at discharge was two.

### Case B

**Figure 2:**
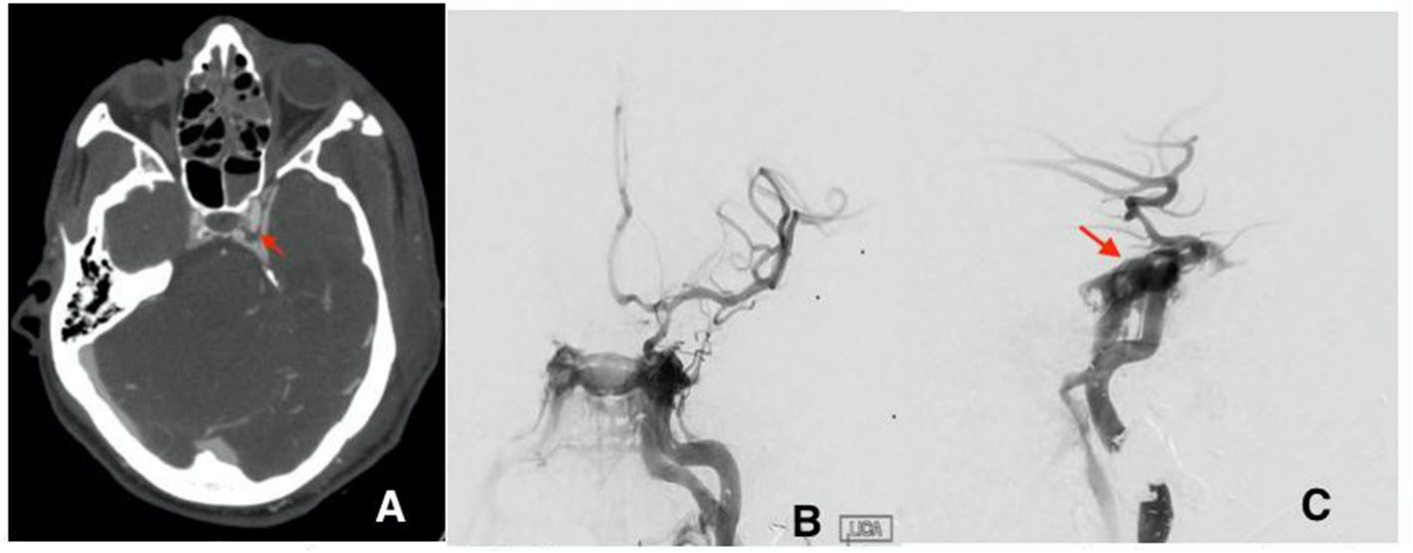
**(A)** Initial CTA with left cavernous pseudoaneurysm (red arrow). **(B)** first cerebral angiogram AP view and lateral view **(C)** showing the direct fistula between the cavernous segment of the left ICA and the left cavernous sinus at the site of ruptured pseudoaneurysm.

**Figure 3:**
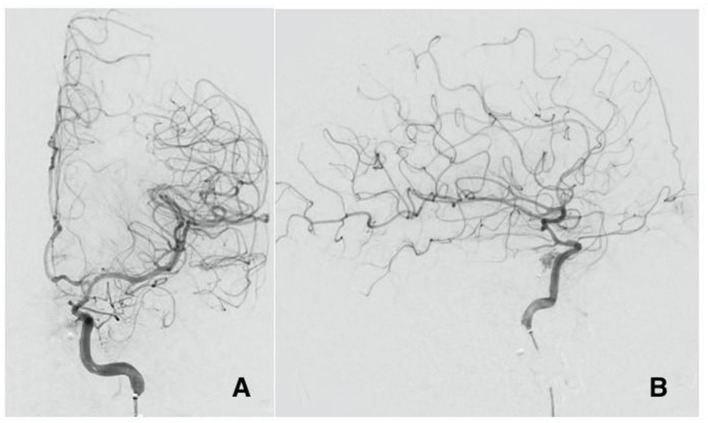
Cerebral angiogram AP view **(A)** and lateral view **(B)** post flow diverter stenting at the site of the ruptured pseudoaneurysm with significant decrease of the flow in the fistula and improved perfusion to the brain.

**Figure 4:**
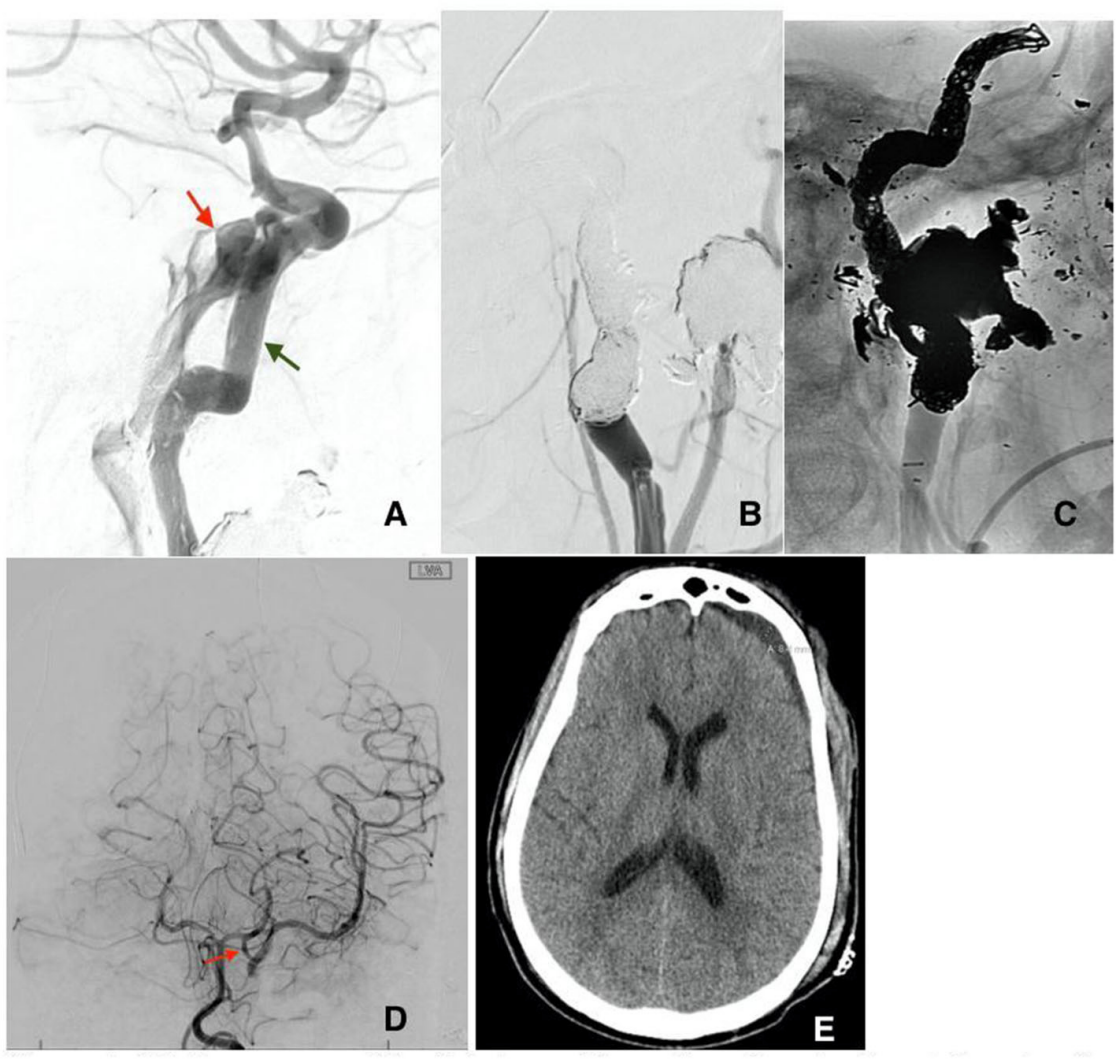
**(A)** Recurrence of the fistula post flow diversion stenting at the site of the ruptured pseudoaneurysm (red arrow). Notice the misplacement of the previously deployed stents (green arrow) secondary to the high flow of the CCF. Subtracted **(B)** and unsubtracted **(C)** views of the left ICA post sacrifice by means of coiling without any residual flow in the CCF. **(D)** Cerebral angiogram of the left vertebral artery post left ICA sacrifice AP view, showing adequate flow into the left middle cerebral artery through a large left posterior communicating artery (red arrow). **(E)** Head CT before discharge showing residual subdural hematoma with no signs of ischemic changes in the left hemisphere.

Patient B is a 30-year-old male who presented at the emergency department after a gunshot wound to the left side of the head. Initial GCS was 8 (E2, V1, M5), and the patient was intubated for airway protection. HCT suggests the bullet took a left antero-posterior trajectory with an entry site below the left orbit, dural penetration, and retained bullet fragments in the temporal lobe. In addition, the patient suffered a left hemispheric subdural hematoma, left temporal lobe injury, and diffuse traumatic subarachnoid hemorrhage. The injury also resulted in a temporal bone fracture, lateral to the carotid canal, extensive left facial fractures including the left zygomatic arch, left maxilla, sphenoid left pterygoid process and body, hard palate, left mandibular ramus, and temporomandibular joint as well as left mastoid and left external auditory canal. A CTA of the head and neck revealed mild irregularity of the intracranial left ICA at the carotid canal. A total of 3 days from presentation, CTA of the head and neck was repeated revealing a new 4 mm pseudoaneurysm of the cavernous segment of the left internal carotid artery (Figure 2A). Conventional cerebral angiography revealed the presence of a high-flow left tCCF with angiographic evidence of compromised flow intracranially (Figures 2B,C). A balloon test occlusion (BTO) was considered. However, blood pressure reduction preceding balloon inflation was associated with loss of somatosensory potentials over the left hemisphere. BTO was subsequently aborted and blood pressure was optimized to ensure appropriate perfusion of the left hemisphere. Following a multidisciplinary discussion, the decision was made to attempt repair of the tCCF with a flow diverting stent that may optimize blood flow to the compromised hemisphere and allow for a proper BTO should that be needed. Following deployment of the stent, improved flow to the left hemisphere was immediately noted (Figures 3A,B). The patient was started on dual antiplatelet therapy for stent patency. The patient later on developed a CSF leak that necessitated an extensive surgical repair that would not have been possible while on dual antiplatelet therapy. In an attempt to find a therapy that spares dual antiplatelet therapy, the decision was made to repeat the BTO in preparation for a possible left internal carotid artery sacrifice at the site of the tCCF. The patient passed the BTO and the left ICA was successfully embolized from the cavernous segment distally into the distal cervical segment proximally with no evidence of any flow into the carotid-cavernous fistula (Figures 4A–C). Dual antiplatelet therapy was discontinued and the patient was maintained on a low dose of aspirin. Facial fractures and CSF leaks were repaired. The patient's length of stay at the hospital was 27 days and he was discharged to an acute rehabilitation facility with no left hemispheric deficits (Figures 4D,E). His GOSE at discharge was five.

## Discussion

Traumatic carotid-cavernous fistulas after gunshot wounds to the head have been described in limited case series (13, 14, 18). Currently, no substantial data exists on ideal screening modality or timing of screening, ideal approach and timing of repair, or possible complications related to interventions (17). Oftentimes, patients who suffer gunshot wounds to the head may incur concomitant intracranial vascular injury, suffer coagulopathy, and need surgery for cranial or other traumatic injuries (14, 17, 19); yielding an inherent challenge to the potential use of dual antiplatelets often utilized in the setting of flow diverting stents. Furthermore, the acute nature of the tCCF and the vulnerability of the acutely traumatized brain renders therapeutic occlusion of parent vessels at a higher risk of ischemic stroke. Therefore, managing tCCF in these patients is oftentimes more complex as compared to tCCF not associated with gunshot wounds.

Risk factors associated with the development of tCCFs appear to be similar to other cerebrovascular injuries; they include the frontobasal site of entry and evidence of injury in the proximity of the cavernous sinus (20). As a matter of fact, the penetrating nature of gunshot wounds causing tCCF is, arguable, not unique. In the context of seemingly blunt trauma, basilar fractures may be associated with a penetrating mechanism causing vascular disruption (21). Gunshot wounds represent the end of a spectrum of injuries associated with tCCF (17). On one end of the spectrum are blunt injuries with no clear evidence of bony fracture that may directly compromise the integrity of the vessel, causing in most cases a low-flow type of tCCF, and on the other end are gunshot wounds where the bullet trajectory may include a direct disruption to the vessel wall, cavernous dura and the bony structures surrounding it. The latter is mostly associated with high flow, or type A, tCCF. Within this continuum are blunt injuries with extensive skull base fractures where the bone fragmentation may represent a penetrating mechanism (4–6).

Screening for vascular injuries, in general, should be considered in any patient with gunshot wounds to the head (14, 17). Our cases demonstrate that the development of tCCF may occur rapidly after the trauma, and screening for cerebrovascular injury, after hemodynamic stabilization is achieved, should be considered in the acute setting. While imaging done at presentation may not be diagnostic, repeat imaging within the first 72 h may reveal previously occult injuries. This is particularly important when the bullet path or associated fractures suggest injury to a particular vascular structure (20). In the cases described above injury to the carotid canal and carotid artery was identified as high flow tCCF. The development of tCCF can occur due to an initial direct injury to the carotid or a result of indirect blast injury. CTA of the head and neck has been shown to perform with good sensitivity in detecting intracranial arterial injury in the carotid artery and first branches of intracranial arteries and, if performed in the hyper-acute phase (immediately on presentation), may detect the presence of a pseudoaneurysm before the development of tCCF (15, 22). However, metallic fragments in the proximity of the cavernous sinus may obscure the presence of arterial injuries. Therefore, when clinical suspicion exists, conventional diagnostic angiography should be considered (22). In the event that hemodynamic stability or clinical suspicion do not justify early conventional angiography, repeating CTA head and neck within 3–7 days from injury may reveal an evolving vascular injury better characterized at that time.

The typical clinical presentation of tCCF, characterized by proptosis, chemosis, orbital bruits, headache, stroke signs, and visual disturbances, is unreliable in this patient population given the critical illness, instrumentation, concomitant globe and facial injury, and compromised level of consciousness (14). Therefore, the investigation of tCCF should not be solely based on clinical presentation.

Prior to definitive treatment of tCCF, optimizing blood pressure goals is advisable in this patient population. Adequate reduction of blood pressure may be needed to prevent significant shunting from the arterial to the venous systems, causing venous engorgement, parenchymal injury, intraparenchymal hemorrhages, and intracranial hypertension (9). However, overzealous reduction in blood pressure may be associated with a further reduction of blood flow to the ipsilateral hemisphere ultimately compromising cerebral perfusion. Therefore, a specific blood pressure target is difficult to establish, and continuous neurological monitoring, whenever possible, is crucial. That being said, neurological assessments are usually limited in this particular cohort of patients thereby clinically limiting examination-based assessment of hemispheric perfusion. Alternative monitoring methods include invasive (LICOX) or non-invasive such as (Near-infrared spectroscopy) tissue oxygenation monitoring, quantitative electroencephalography (q-EEG), and transcranial doppler (TCD), may provide insight while blood pressure manipulation is underway (23–26).

Repair is advised as soon as possible, hemodynamic status permitting. Ideally, repair of tCCF, or any other cerebrovascular injury, should precede less urgent surgeries, such as facial or ophthalmological intervention. An exception to the above is life-saving procedures, such as emergent decompressive craniectomy in patients with uncontrolled intracranial hypertension or evolving intracranial compartment syndrome, as described in our first case.

A multidisciplinary team approach including neurointensive care, vascular neurosurgery, and neuro-endovascular surgery is advised in these complex patients. Endovascular treatment is nowadays the preferred therapeutic approach for tCCF (10, 11). The repair can occur by means of embolization of the fistula or placement of flow-diverting stents (27, 28). Very importantly, stent placement requires the initiation of antithrombotic therapy to avoid stent thrombosis. Therefore, stenting should be cautiously considered in GSW patients. In fact, the use of antiplatelet therapy may be contraindicated in presence of intracranial hemorrhage, need for additional surgical interventions, placement of intracranial monitoring, or lumbar drainage. Newer flow-diverting stent technologies necessitating single antiplatelet use are a promising advancement. When repair of the fistula is not possible or unsuitable due to the patient's clinical condition, the sacrifice of the internal carotid artery is a last viable therapeutic resort, ideally after demonstrating adequate collateral cerebral perfusion. In patients with GSW to the head, the presence of vascular injury has been shown to be associated with worse outcomes (14). However, the specific association between tCCF and outcome remains less certain.

## Conclusion

Traumatic CCF may occur in patients with gunshot wounds to the head, representing an extreme of penetrating mechanisms associated with this type of injury. Current penetrating brain injury guidelines are outdated and provide no consensus on the management of this condition. If possible, immediate and early cerebrovascular imaging is the preferred screening modality in this patient population as relying on clinical manifestations is usually limited by poor neurological status and possibly concomitant injury. Blood pressure control is an important step in the management and should ideally balance between brain perfusion and minimizing flow across the fistula. Definitive repair should be attempted as soon as medically possible. Embolization of the fistula, flow diversion via stenting of the fistula site, and, finally, vessel sacrifice are viable options depending on the size of the fistula, flow grade, collateral flow, phase on injury, and concomitant injury that may dictate permissibility of antithrombotic therapy.

## Data Availability Statement

The original contributions presented in the study are included in the article/Supplementary Material, further inquiries can be directed to the corresponding author.

## Ethics Statement

Written informed consent was obtained from the individual(s) for the publication of any potentially identifiable images or data included in this article. Written informed consent was obtained from the participant for the publication of this case report.

## Author Contributions

All authors listed have made a substantial, direct, and intellectual contribution to the work and approved it for publication.

## Conflict of Interest

The authors declare that the research was conducted in the absence of any commercial or financial relationships that could be construed as a potential conflict of interest.

## Publisher's Note

All claims expressed in this article are solely those of the authors and do not necessarily represent those of their affiliated organizations, or those of the publisher, the editors and the reviewers. Any product that may be evaluated in this article, or claim that may be made by its manufacturer, is not guaranteed or endorsed by the publisher.
